# The inclusion of psychological factors in the evaluation of a curriculum enrichment program for students with high ability

**DOI:** 10.3389/fpsyg.2025.1699593

**Published:** 2025-10-22

**Authors:** Leire Aperribai, Garbiñe Moreno, Ainize Sarrionandia, Karmele Salaberria

**Affiliations:** Department of Clinical and Health Psychology and Research Methodology, Faculty of Psychology, University of the Basque Country UPV/EHU, Donostia-San Sebastián, Spain

**Keywords:** academic achievement, creativity, enrichment program, evaluation, learning approach, self-esteem

## Abstract

There is currently evidence of the importance of some psychological factors in long-term academic performance. In this study, the impact that a curriculum enrichment program has had on academic performance has been evaluated considering psychological variables related to academic performance. A quasi-experimental study of two groups (experimental and control), based on a design of repeated measures was carried out. Participants were students with high ability of 5 and 6 years of Primary Education and 1 and 2 years of Secondary Education of 9 educational centers, aged between 9 and 13 years old, being 68% boys and 32% girls. Psychological variables such as self-esteem (Rosemberg Self-Esteem Scale), learning approach (Revised Survey of Study Processes), and creativity (Test CREA), as well as academic achievement or performance (academic scores) were assessed to evaluate the curriculum enrichment program Sakonduz. Results show that, in respect to academic achievement, the experimental group unexpectedly worsened while the control group improved, but in both cases the differences were not significant. As for the learning approach, the control group didn't show any changes, as expected, while the experimental group maintained the deep motivation approach (DM) and decreased the deep strategy approach (DS), and consequently the deep learning general approach (DA). Also, the results showed a considerable decrease in the level of self-esteem of the control group, while it was maintained at the same high level in the experimental group. Finally, creativity measurements didn't have consistent results. This curricular enrichment program should be implemented as a long-term and more intense program to obtain more evidence of its effect on psychological aspects. Nevertheless, an important attrition occurred, and it would be important for the future the implication of the schools and teachers to implement and collect more evidence of the program's effectiveness.

## 1 Introduction

Curricular enrichment programs directed to students with high ability are part of the educational response that should be offered by educational systems. Even when they remain still far from the optimal application, these programs are necessary to promote learning processes. However, on the one hand, in the cases that these programs are carried out by educational institutions, some of these are not usually evaluated, or they are valued only by considering the academic performance of the students. Thus, sometimes it is not known if the programs have an impact on the students. On the other hand, there is currently evidence of the importance of some psychological factors in long-term academic performance, so that they should be included in the evaluation of these programs. In this study, the impact that a curriculum enrichment program has had on academic performance has been evaluated considering psychological variables that have received scientific evidence of their relationship with academic performance. Factors such as self-esteem, the learning approach (related to motivation and strategies), and creativity have been included.

The concept of high ability has had an evolution, so that it has been understood from a static intelligence to a dynamic and developable concept, in which factors such as heritage, environment and personal qualitative aspects (motivation, perseverance, or effort) influence. Thus, abilities are irregular and can change over time and different stages of life (Aretxaga, 2013).

Even when there are numerous and varied definitions of high abilities, partly because this population is composed of a very heterogeneous group, the current paradigm understands this construct in a multidimensional way and proposes that it is expressed in different profiles (talents or giftedness). From a neuropsychological perspective, high abilities are understood in terms of brain configuration and functioning, so that highly able people have greater neural efficiency, as their brain selectively and simultaneously activates the areas necessary to solve the task, with greater myelination and supposing lower cortical metabolic consumption (Sastre-Riba, 2011). The functional differences of this group are based in characteristics such as an exceptional competence, a high speed of learning, precocity, as well as a capacity, flexibility, and effectiveness to understand and solve complex problems (Rodríguez-Naveiras et al., 2019). Thus, during childhood and adolescence, they have potential above the expected means at their age, which may or may not develop, in interaction with the environment. Highly able students present singular characteristics such as a great curiosity, ability to reason in a complex way, accuracy and richness of language, symbolic or abstract thinking, good long-term memory, fast learning, great creativity and imagination, precocity and/or desynchrony between different areas of development, high emotional sensitivity, early concern for social issues, a sense of elaborated humor, broad interests, intrinsic motivation and independence of thought, performance of tasks and trend.

The educational needs of these students aim to develop their potential for excellence. For that purpose, not only should their capacities and objective features be considered, but also factors such as motivation, temperament, perfectionism, desynchronies or other environmental factors (family, school, or social factors) must be taken into account (Borges and Rodríguez-Naveiras, 2012; Gobierno Vasco – Eusko Jaurlaritza, 2014, 2019). Thus, highly able students have specific needs for educational support, these being intellectual, social and emotional needs.

Aretxaga (2013) defines curricular enrichment as an ordinary educational measure (applied by law) directed to students identified by the educational system as highly able students, in which it carries out an extension and/or deepening of the curriculum, whose purpose would be to respond to the needs of the students based on the development of their capacities, and oriented toward content, process and product. This measure allows highly able students to continue in their ordinary classroom, so that they can continue sharing the space and experiences with their peer group while developing intellectually with the supervision of a tutor or teacher. But it can also be implemented through programs in heterogeneous and flexible clusters, composed of students of different ages. The measure benefits highly able students by adapting the curriculum to the students' educational needs (mainly cognitive, motivational, and emotional needs) and makes possible addressing different modalities such as compaction of the curriculum (both eliminating known content and adding new ones), the in-depth enrichment (of existing content), or autonomous work on themes or projects that are not within the curriculum of the course, although they are directly related (Gobierno Vasco – Eusko Jaurlaritza, 2014).

There are many opportunities offered by curriculum enrichment programs. However, (Borges and Rodríguez-Naveiras 2012) stress the need for an evaluation inherent to these programs in order to assess the results of the intervention, through the application of systematized procedures. Thus, a rigorous evaluation provides answers on the functioning and effectiveness of the program, enables quality improvement and also identifies possible unobserved effects that may be harmful. These authors carried out a review of different studies that evaluated programs for highly able students and observed that they all had positive effects. However, it has been observed that there are numerous factors to be considered, such as identification, curriculum, program design, implementers, and parental involvement. But also, the personal or psychological factors of the students, such as self-esteem, motivation or the possibility to develop creativity can influence the results of these programs. This is an aspect that few studies consider (Kim, 2016), because many times psychological variables have been forgotten when evaluating enrichment programs.

A learning approach is based on a motive or intention that directs the students toward a series of learning strategies. Thus, it is understood that the student has relatively stable motives for the work he/she performs, given that he/she has a conception about what academic learning should be. This leads the student to develop the learning process in a consistent way. This consistency of motives and strategies is what Biggs calls learning approaches (Hernández-Pina et al., 2004). A lack of consistency may lead to poor academic performance.

Given the goals that the student has to achieve and after a period of exposure to a specific teaching/learning framework, the student's self-perception of his/her ability, the teaching method and the evaluation, the results obtained, etc., will help him/her to develop a certain approach, superficial or deep, to enable him/her to carry out the academic tasks as comfortably as possible. These two approaches are the effect of meta-learning, although they may act independently of metacognitive processes. Thus, students with a low level of cognitive sophistication often resort to a superficial approach where the use of strategies is done mechanically (Hernández-Pina et al., 2004).

Self-esteem is the evaluative dimension of self-concept, it indicates the person's appreciation of himself/herself, and it carries the values placed in the self (Cid-Sillero et al., 2020). Self-esteem has not always been distinguished from self-concept, but even when this is a similar construct, there is an important difference between them. Self-concept refers to the idea that each one has about himself/herself while self-esteem is the evaluation of self-concept. Self-concept is a multidimensional construct with an organized structure that separates the academic and non-academic aspects, the latter being also divided into the physical, social and personal dimensions. In addition, it is considered stable, although its multidimensionality increases with the person's lived experiences. In contrast, self-esteem refers to the subjective perception of one's own self-worth, the individual's feelings of self-respect and self-confidence, and the individual's positive or negative views about itself (Sedikides and Gress, 2003). In the educational context, self-esteem can favor the understanding of the students‘ behavior, as it has been seen that students' beliefs about their abilities have a greater impact than the capacity itself in academic performance. Thus, it has been observed that self-esteem and school performance are positively related (Cid-Sillero et al., 2020). So that low self-esteem may influence negatively on academic performance.

Obviously, self-esteem is also important for highly able students, since different studies have shown that enrichment programs benefit their self-concept (Feldhusen et al., 1990) and their self-esteem (Hertzog, 2003). Simple participation in a program, the possibility to perform difficult tasks and to work hard, the opportunity to challenge themselves and to develop their potential, and the achievement of the challenges proposed in these difficult tasks, make them enjoy and feel well with their learning and education. Gubbels et al. (2014) consider that having a positive self-concept is related to higher performance, so an enrichment program should effectively improve both cognitive and socioemotional characteristics in highly able students. These authors confirmed this idea in a study in which they found that the enrichment program favored the improvement of intelligence skills, as well as their self-concept, in highly able students of basic level of education.

Creativity is often defined as the ability to generate new and effective ideas (Gubbels et al., 2022), and its process involves being sensitive to problems, fluidity of ideas, potential to change perspective, tendency to give an individual and original response and ability to redefine and interpret (Özgenel et al., 2019). In addition, Orozco-Gómez et al. (2023) define creativity as a complex multidimensional concept that goes beyond the definition proposed above. In addition to generating new ideas, creativity also plays a crucial role in problem solving, critical thinking and finding solutions to daily problems. In this same line, Zhu et al. (2019) find that an important predictor for creativity is divergent thinking, which is in turn moderated by convergent thinking.

Creative people aren't necessarily skilled at doing conventional tests to measure intelligence, because they can solve problems differently than expected. These people demonstrate great flexibility and ability to adapt, intuition and insight. But those who generate new quality ideas also need analytical capacity to evaluate their effectiveness and be able to use them. So creative highly able students may also benefit from practical intelligence to put their ideas into action (Aljughaiman and Ayoub, 2012), as they could produce original ideas, and could improve motivation and engagement in the highly able students, as active educational strategies may have these effects in these students (Aretxaga, 2013). Moreover, currently creativity is very much in demand in the labor market, so it is considered necessary to approach it in the educational system and learn more about the effects of extracurricular activities in this construct (Orozco-Gómez et al., 2023).

Studies evaluating enrichment programs have yielded a positive result in increased creativity, namely an improvement in creative and analytical capacity (Aljughaiman and Ayoub, 2012), capacity building and skill development through creative thinking and problem solving (Nogueira, 2006), an increase in creative skills after programs (Özgenel et al., 2019) and a positive effect on the creativity of students with high abilities through an out-of-class program (Orozco-Gómez et al., 2023). However, some studies have found no effect of the program on creativity, as is the case of Gubbels et al. (2022), whose assessment of an enrichment program had a stable improvement in creative skills based on pre-intervention capacity, but not due to the same intervention.

Current educational legislation in the context of this research establishes that students with high abilities should receive action plans and enrichment programs to meet their needs and achieve optimal development, adapting education to their needs. Within the framework of an inclusive school, the response to the diversity and the specific needs of the students must be met in a standardized and inclusive environment, considering their interests, motivations and learning capabilities. Another proposal to intervene with this population is the individualized action plan, within the educational intervention plan for students with high intellectual abilities (Gobierno Vasco – Eusko Jaurlaritza, 2019), which seeks the integral development of these students. It is proposed to work on self-esteem, care, the ability to plan and manage work, the study habits and barriers that may exist in the classroom. It also seeks to involve families in the improvement of their children.

Despite legislative progress, there are still several barriers to its proper implementation, such as difficulties in detecting, implementing individualized action plans and implementing enrichment programs, due to myths and stereotypes or lack of resources, training and evaluation (Casino-García et al., 2019; Ersoy and Uysal, 2018). These difficulties have considerable consequences for all those involved in the educational process, both students, teachers and parents. For their part, students do not achieve significant learning, which can lead to low academic performance, difficulties to develop creativity, low satisfaction, loss of self-esteem and lack of motivation (Casino-García et al., 2021; Godoy, 2017; Lamanna et al., 2019). Teachers, in turn, may feel frustrated, demotivated and not involved (Matheis et al., 2017). Parents, in addition to frustration, may feel hopelessness, anger or anxiety about the situation (Rodríguez-Naveiras et al., 2019).

While enrichment programs are an ordinary measure that schools must carry out, there are few studies on their evaluation and little information on their effectiveness. Most of the extracurricular enrichment programs are evaluated, especially through universities. However, there is a lack of data that sheds light on the evaluations of curricular enrichment programs implemented by educational administrations. Only in a few cases have these been evaluated (mainly by universities), but, above all, academic performance or cognitive factors are considered as the main evaluation criterium. In this line, Kim (2016) carried out a meta-analysis to analyze the effects of enrichment programs on students with high abilities. Among the 26 studies analyzed, some considered programs included in the academic year and other programs applied in summer activities. In addition, some analyzed academic performance as an evaluation criterion, while others analyzed socio-emotional outcomes. In the latter, only 3 studies out of 26 total analyzed self-concept and 1 of them with repeated measures pre-post. None analyzed the learning approach or motivation as such, although 1 study analyzed the intrinsic value and two other attitudes toward learning. Considering the importance of these factors in significant learning and learning outcomes, it would be interesting to consider these aspects in the evaluations of these programs. This is in accordance with Aljughaiman and Ayoub (2012), who also noted that studies assessing the effects of enrichment programs tend to focus only on traditional variables such as cognitive ability, motivation, academic performance, learning attitudes and behavioral improvement.

In one of the studies considering psychological variables to evaluate enrichment programs, these have been found to be important. Thus, (Casino-García et al. 2021) have found that these programs entail improvements in not only school performance, but also contribute to the wellbeing of students, motivation, self-concept, interpersonal relations and conflict resolution. Other studies conclude that participation produces positive effects at both academic and personal level (Aljughaiman and Ayoub, 2012; Rodríguez-Naveiras et al., 2019). In addition, Gubbels et al. (2022) have observed that curricular enrichment programs prevent poor performance and school failure in highly able students.

As previously observed, there are few studies evaluating enrichment programs in students with high ability, and fewer studies consider psychological variables in the evaluation, besides measuring school performance (Kim, 2016). Therefore, this research is expected to contribute to the improvement of these programs and highlight the need to include psychological factors such as self-esteem, the learning approach or the motivation and creativity in their evaluations. The objective of this study is to evaluate the effectiveness of the Sakonduz curriculum enrichment program, by considering the psychological variables of self-esteem, the learning or motivation approach, and creativity. We hypothesize that the Sakonduz program will improve many academic and personal aspects in highly able students: (1) it will improve the academic achievement of the students; (2) it will contribute to the increase of a deep learning approach and the decrease of a superficial learning approach; (3) it will improve their self-esteem; (4) it will foster the development of creativity of students. Also, we hypothesize that: (5) there will be a positive relationship between academic achievement, deep learning approach, self-esteem and creativity and a negative relationship between the previous variables and superficial learning approach.

## 2 Method

### 2.1 Research design

The study was based on a quasi-experimental design of two groups, with independent measures in the intervention factor. Both groups participated in a pre- and post-program evaluation; a design of repeated measures was carried out. The experimental group participated in the Sakonduz curricular enrichment intervention program, while the control group did not participate in any such program.

### 2.2 Participants

This study involved students with high ability of 5 and 6 years of Primary Education and 1 and 2 years of Secondary Education, aged between 9 and 13 years old who belonged to 9 centers in the province of Gipuzkoa, 2 of them public and 7 concerted. The groups were formed by the Aupatuz association, which was responsible for the Sakonduz project in its entirety. The inclusion criterion for the experimental group was that the students should participate in the Sakonduz curriculum enrichment program for students with high ability; as for the control group, the only inclusion criterion was that they should have an evaluation of high ability. Thus, all participants were identified as highly able students in the public educational administration by following the official protocols established by the government. At the beginning, there were 72 students who started participating in the study: from those, 47 were assigned to the experimental group (participants of the Sakonduz program), 33 boys (70.21%) and 14 girls (29.79%); and 25 participants were included in the control group, 17 boys (68%) and 8 girls (32%). Due to the experimental mortality at the end of the school year and the timing of the intervention, only data from 3 of the 7 concerted centers could be collected and, except for academic performance data, only 25 students were kept for the rest of the measures: from those, 16 belong to the experimental group, 9 boys (56.25%) and 7 girls (43.75%); and 9 to the control group, 8 boys (88.89%) and 1 girl (11.11%). This final group was aged between 10 and 13 years (*M* = 11.60; *SD* = 0.82). Thus, the percentage of abandonment of the experimental group was 65.96%, while in the control group it was 64.00%. Finally, only 22 participants responded in the second measure of the Creativity test. This high attrition rate in the experimental group was due mainly to the lack of involvement of the educational centers. As for the control group, the main reason was related to the loss of interest among the participants.

### 2.3 Instruments

The study analyzed variables that were considered important for the evaluation of the curriculum enrichment program Sakonduz: self-esteem, learning approach (motivation and strategies), creativity and academic achievement or performance. To evaluate the variables mentioned, the instruments below were used.

#### 2.3.1 Rosemberg self-esteem scale (Atienza et al., 2000)

The students' general self-esteem was evaluated through this scale, specifically with the adaptation made to Spanish by Atienza et al. (2000) of the Rosenberg Self-esteem Scale or EAR (1965). The scale consists of 10 items, 5 of them inverted, with 4-point Likert-type responses, ranging from 1 (“Strongly Disagree”) to 4 (“Strongly Agree”); the total score is between 10 and 40 points. Self-esteem is measured in a one-dimensional way, although some authors propose a two-factor structure, considering both positive and negative self-esteem. The scale has shown good reliability index with Cronbach's alpha of 0.86 (Atienza et al., 2000).

#### 2.3.2 Revised survey of study processes (Recio and Cabrero, 2005)

To measure the learning approach, the revised version of the Study Process Questionnaire of 2 Factors (R-SPQ-2F; Biggs et al., 2001), in the Spanish version by Recio and Cabrero (2005) was used. It consists of 20 items, on a 5-level Likert scale, ranging from 1 (“Never or Almost Never”) to 5 (“Always or Almost Always”). The items are divided into two general factors, both of 10 items: Deep Learning (DL), subdivided into Deep Motivation (DM) and Deep Strategy (DS); and Superficial Learning (SL), subdivided into Superficial Motivation (SM) and Superficial Strategy (SS). In the adaptation of the scale, the DA dimension obtained a Cronbach Alpha of 0.87 and the SA dimension a Cronbach Alpha of 0.75.

#### 2.3.3 Test CREA (Corbalán et al., 2003)

The instrument used to measure creativity was the CREA Test, specifically the sheet C of this test, aimed at boys and girls aged 6 years or older. The participants were asked to ask as many questions as possible in relation to the image of sheet C, in a limited time of 4 mins. Each question demonstrates the ability to create a new idea, resulting from the interaction between the stimulus and an existing cognitive scheme. As for the correction, questions may be valid, invalid or receive an additional score if the question consists of more than one scheme or content. The manual presents standardized scores for the Spanish and Argentine populations. In the Spanish version, the responses to the sheet C could have a score range between 0-25 points, being the mean 9.35 (*SD* = 5.74) for scholars between the age of 6-11 years and 11.47 (*SD* = 4.67) for scholars between the age of 12-16 years. In this study, two people were responsible for correcting and agreeing on the scores that were subsequently analyzed.

Finally, school performance was assessed through the final grades the participating students obtained in the last two courses.

### 2.4 Procedure

Sakonduz is a curriculum enrichment program carried out by the Aupatuz association. Briefly, Aupatuz is an association composed of families with highly able children, and its main objective is to achieve the emotional wellbeing of their children, seeking a balance between intellectual, emotional and social development. They work and collaborate with different educational actors to achieve an inclusive school that takes into account the needs of students with high skills. The Sakonduz project was designed as an online extracurricular enrichment program, aimed at students of fifth and sixth grade of Primary Education and first and second of the Secondary Education that needs this service. Its duration is that of a school year and is designed for the student to dedicate 2 h a week, one in the classroom and one at home. It consists of four modules: mathematical thinking, literary creation in Basque language, philosophy and sciences. Each module is carried out by a specific teacher, whose function is to develop materials based on appropriate pedagogical criteria for students. They are taught bilingual, in Basque (local language of the Basque Country) and in Spanish (national language). Everyone's goal is to work on things like creativity, critical sense, imagination, and positive self-concept. A pilot test was carried out for their integration into the curriculum. In its second year in operation, it has been applied as a curriculum enrichment program in 9 educational centers in the province of Gipuzkoa, where highly able students of the mentioned centers and grade levels took part as school-time activities integrated into the different subjects. This study gathers the results of the evaluation that has been carried out to check its effectiveness.

In order to carry out this evaluation, once the evaluation criteria were established, we proceeded to make an online questionnaire that could be integrated into the Sakonduz educational intervention web program. Then we proceeded to request approval from the Ethics Committee of the University of the Basque Country UPV/EHU (CEISH-UPV/EHU; M10_2022_282). The sampling was carried out through the Aupatuz association, since it was the entity that was in contact with the educational centers. Once all the permits were obtained, data were collected prior to the Sakonduz intervention program (October 2023). Finally, the measures were repeated at the end of the program (May-June 2023).

### 2.5 Data analysis

Quantitative data were analyzed through descriptive data analysis (including asymmetry and kurtosis analysis) Cronbach's alpha and test-retest reliability estimates. Differences between averages (ANOVA for repeated measures), along with the Eta partial squared effect sizes were analyzed for learning approach dimensions and self-esteem. In addition, due to the non-compliance with the assumption of normality, for the creativity and academic performance or achievement variables, analyses of median differences were also performed using the non-parametric Wilcoxon sign range test for related samples. Finally, Pearson correlations between academic achievement, learning approaches, self-esteem and creativity were analyzed. The analyses were performed using IBM-SPSS v29.

## 3 Results

### 3.1 Academic achievement

#### 3.1.1 Descriptive data

The experimental group (*N* = 6) obtained a median score of 9.00 (*SD* = 0.00) out of 10 in the year before the enrichment program, to the same as the score of 9.00 (*SD* = 0.98) in the subsequent year. The control group (*N* = 6) obtained a median score of 9.00 (*SD* = 0.68) out of 10 in the year before the enrichment program, compared to the score of 8.65 (*SD* = 0.96) in the subsequent year.

#### 3.1.2 Reliability

The result obtained by the test-retest measurement indicated the existence of a median reliability index based on the stability between both measures in the experimental group (*r* = 0.551; *p* = 0.012), as in the control group (*r* = 0.810; *p* = 0.003).

#### 3.1.3 Differences between pre- and post-program measures

The Shapiro-Wilk normality tests indicated that this assumption was not met both for the previous measurement in the experimental group [S-W (20) = 0.236; *p* < 0.001], as for the measurement in the control group [S-W (11) = 0.782; *p* = 0.005]. Since the assumption of normality was not met, an analysis of median differences was performed using the non-parametric Wilcoxon signed range test for related samples. The results showed that there were no significant differences between the two performance evaluations either in the experimental group (*W* = 6; *p* = 0.090), or in the control group (*W* = 10.5; *p* = 0.288).

### 3.2 Learning approach

Differences between means of repeated measures of Deep Motivation (DM) and Deep Strategy (DS), Superficial Motivation (SM) and Superficial Strategy (SS), as well as general measures of Deep Learning Approach (DA) and Superficial Learning Approach (SA) were analyzed.

#### 3.2.1 Descriptive data

In the first approximation, the means and standard deviations, as well as asymmetry and kurtosis statistics were analyzed in the two repeated measures of the different variables for the experimental (see Table 1) and control (see Table 2) groups. Regarding the distribution statistics, these showed adequate values in both groups, showing absolute values of asymmetry below 2 and of kurtosis below 7 (Curran et al., 1996).

**Table 1:** Means and standard deviations, and asymmetry and kurtosis statistics of pre- and post-test measures of learning approach in the experimental group.

**Approach**	**Pretest**				**Posttest**			
	**M**	**DE**	**Asymmetry**	**Kurtosis**	**M**	**DE**	**Asymmetry**	**Kurtosis**
DM	17.50	4.09	−0.673	−0.253	14.50	4.44	−0.482	−1.700
DS	18.17	4.49	−0.164	2.048	15.75	3.86	0.169	−4.409
SM	10.67	3.88	0.193	−1.354	10.75	3.59	−0.889	−0.582
SS	12.33	3.50	1.763	3.559	13.50	5.75	−0.517	1.649
DA	35.67	7.97	−1.062	0.884	30.25	6.70	−1.059	2.042
SA	23.00	6.42	0.565	−0.489	24.25	7.93	0.413	−1.667

DM: Deep motivation; DS: Deep strategy; SM: superficial motivation; SS: superficial strategy; DA: Deep learning approach; SA: superficial learning approach.

**Table 2:** Means and standard deviations, and asymmetry and kurtosis statistics of pre- and post-test measures of learning approach in the control group.

**Approach**	**Pretest**				**Posttest**			
	**M**	**DE**	**Asymmetry**	**Kurtosis**	**M**	**DE**	**Asymmetry**	**Kurtosis**
DM	17.83	2.48	0.871	0.735	18.17	2.93	0.330	−2.192
DS	18.50	3.21	−0.191	−1.305	17.33	2.34	−0.600	−1.289
SM	9.67	1.97	−0.254	−1.828	9.33	3.78	1.183	1.815
SS	12.33	2.73	−0.435	0.586	11.50	3.73	−0.486	−1.546
DA	36.33	3.72	−1.125	0.586	35.50	4.68	−0.141	−2.843
SA	22.00	3.80	−1.548	2.459	20.83	6.65	0.057	1.025

#### 3.2.2 Reliability

Cronbach's alpha indices for the whole sample in the pretest measures revealed fair but acceptable internal consistency of the dimensions (see Table 3), considering Nunally (1978) criterion. The result obtained by the test-retest measurement indicated the existence of good and bad reliability estimates based on the stability between the repeated measurements of the different approaches in both the experimental and the control group (see Table 3). In the experimental group only the Pearson correlations of the repeated measures of the deep approaches (DM, DS and DA) were significant, while in the control group only the superficial approaches (SM, SS and SA) correlated significantly.

**Table 3:** Cronbach's alpha of learning approach dimensions for the whole sample and test-retest reliability indices estimated using Pearson correlations for the experimental and control group.

**Approach**	**Experimental group (*****N =*** **16)**		**Control group (*****N =*** **9)**		**Cronbach's alpha (*N* = 25)**
	**r**	**p**	**r**	**p**	α
DM	0.861	0.000	0.102	0.795	0.667
DS	0.804	0.000	0.446	0.228	0.622
SM	0.384	0.142	0.868	0.002	0.648
SS	0.348	0.187	0.778	0.013	0.719
DA	0.923	0.000	0.546	0.128	0.795
SA	0.440	0.088	0.848	0.004	0.804

DM: Deep motivation; DS: Deep strategy; SM: superficial motivation; SS: superficial strategy; DA: Deep learning approach; SA: superficial learning approach.

#### 3.2.3 Differences between pre- and post-program learning approaches

The Shapiro-Wilk normality tests indicated that this assumption was fulfilled (see Table 4). Regarding the supposition of sphericity, the estimate of Mauchly's sphericity test was not considered adequate, since the variables had only two levels, and showed a single covariance that was equal to itself.

**Table 4:** Results of the Shapiro-Wilk normality tests for the pre-test measurements of the learning approach in the experimental and control group.

**Approach**	**Experimental group (*****N*** = **16;** ***df*** = **16)**		**Control group (*****N*** = **9;** ***df*** = **9)**	
	**S-W**	**p**	**S-W**	**p**
DM	0.927	0.221	0.966	0.859
DS	0.954	0.556	0.959	0.793
SM	0.951	0.500	0.908	0.300
SS	0.946	0.423	0.963	0.833
DA	0.930	0.240	0.934	0.518
SA	0.975	0.916	0.903	0.270

DM: Deep motivation; DS: Deep strategy; SM: superficial motivation; SS: superficial strategy; DA: Deep learning approach; SA: superficial learning approach.

In the experimental group, the intra-subject effects tests showed significant differences for the means of DS and DA (in both cases with a decrease in the mean in the second measure, and a large effect size), but not so for the measurements of DM, SM, SS, and SA (see Table 5). In the control group, the intra-subject effects tests showed no significant differences for the means of any of the approach measures (see Table 5).

**Table 5:** Differences between pre- and post-learning approaches and effect sizes in the experimental and control group.

**Approach**	**Experimental group**				**Control group**			
	**F**	**df**	**p**	$\eta_{\text{p}}^{2}$	**F**	**df**	**p**	$\eta_{\text{p}}^{2}$
DM	0.732	1, 15	0.406	0.047	1.149	1, 8	0.315	0.126
DS	4.642	1, 15	0.048	0.236	2.139	1, 8	0.182	0.211
SM	0.038	1, 15	0.848	0.003	0.111	1, 8	0.747	0.014
SS	2.860	1, 15	0.111	0160	0.000	1, 8	1.000	0.000
DA	5.789	1, 15	0.029	0.278	2.573	1, 8	0.147	0.243
SA	1.312	1, 15	0.270	0.080	0.041	1, 8	0.845	0.005

DM: Deep motivation; DS: Deep strategy; SM: superficial motivation; SS: superficial strategy; DA: Deep learning approach; SA: superficial learning approach.

### 3.3 Self-esteem

#### 3.3.1 Descriptive data

The experimental group (*N* = 16) obtained a mean self-esteem of 31.31 (*SD* = 5.30) in the previous measure to the enrichment program, with adequate distribution statistics (asymmetry = −0.114; kurtosis = −1.868), and a mean of 32.88 (*SD* = 5.55) in the post-enrichment program measure, also with adequate asymmetry (-0.068) and kurtosis (-5.348); in both measurements the self-esteem level was high (>30). The control group (*N* = 9) obtained a mean self-esteem of 30.78 (*SD* = 5.59) in the previous measure with adequate distribution statistics (asymmetry = −0.052; kurtosis = −1.874), and a mean of 28.00 (*SD* = 4.64) in the latest measure, also with adequate asymmetry (0.109) and kurtosis (-0.916); the self-esteem level was high in the previous measurement but decreased to an average level (26-29) in the last measurement. The distribution statistics were adequate in both groups, showing absolute asymmetry values below 2 and kurtosis values below 7 (Curran et al., 1996).

#### 3.3.2 Reliability

Self-esteem scale revealed a good internal consistency (alpha = 0.885) for the pretest measure when considering the whole sample. The result obtained by the test-retest measurement indicated the existence of a reliability estimate based in the stability between both measures, both in the experimental group (*r* = 0.827; *p* = 0.000) and in the control group (*r* = 0.816; *p* = 0.007).

#### 3.3.3 Differences between pre- and post-program measures

The Shapiro-Wilk normality tests indicated that this assumption was fulfilled both for the previous measurements in the experimental group [S-W (16) = 0.925; *p* = 0.204] and for the control group [S-W (9) = 0.922; *p* = 0, 413]. Regarding the supposition of sphericity, the Mauchly's sphericity test was considered not to be adequate, since the variables had only two levels, and showed a single covariance that was equal to itself.

In the experimental group, the intra-subject effect test showed that there was no significant difference between the means of self-esteem of the two moments [F _(1, 15)_ = 3.806; *p* = 0.070; η^p2^ = 0.202]. However, in the control group, the intra-subject effect test showed that there was a significant difference between the means of self-esteem of the two moments [F _(1, 8)_ = 6.649; *p* = 0.033; η^p2^ = 0.454], with a decrease in self-esteem between the first and second measurements, and a large effect size.

### 3.4 Creativity

#### 3.4.1 Descriptive data

The experimental group (*N* = 14) obtained a median creativity of 9.50 (*SD* = 3.34; *N* = 16) in the pre-enrichment program measure and 9.50 (*SD* = 5.81; *N* = 14) in the subsequent measure. The control group (*N* = 8) obtained a mean creativity of 12.00 (*SD* = 10.40; *N* = 8) in the pre-enrichment program measure and of 8.00 (*SD* = 5.61; *N* = 8) in the subsequent measure. In all cases, the means showed an average level of creativity (percentile between 26 and 74).

#### 3.4.2 Reliability

The result obtained by the test-retest measurement indicated fair reliability based on the stability of the two measurements in the experimental group (*r* = 0.390; *p* = 0.168), but acceptable in the control group (*r* = 0.772; *p* = 0.025).

#### 3.4.3 Differences between pre- and post-program measures

The Shapiro-Wilk normality tests indicated that this assumption was fulfilled for the previous measurement [S-W (14) = 0.946; *p* = 0.505] in the experimental group. In the control group, however, the assumption was not met [S-W (8) = 0.738; *p* = 0.006].

Therefore, an analysis of median differences was performed using the non-parametric Wilcoxon signed range test for related samples. The results showed that there were no significant differences between the two times in which the creativity test was administered, neither in the experimental group (*W* = 33; *p* = 0.570), nor in the control group (*W* = 1.5; *p* = 0.102).

### 3.5 Relationship between academic achievement, learning approaches, self-esteem and creativity

The results of the experimental group revealed that Deep Strategies (DS) correlated positively and significantly with self-esteem in the pretest measures (see Table 6). But there weren't other significant relationships between the other variables. As for the posttest measures (see Table 7), the same results were found with peculiarity, because self-esteem correlated significantly with both, Deep Strategies (DS) and Deep Approach (DA).

**Table 6:** Pearson's correlations between achievement, learning approaches, self-esteem and creativity for experimental group in pretest measures.

		**Achievement**	**DM**	**DS**	**SM**	**SS**	**DA**	**SA**	**Self-esteem**	**Creativity**
Achievement	Pearson	.a	.a	.a	.a	.a	.a	.a	.a	.a
	p (bilat)									
	N	6	6	6	6	6	6	6	6	6
DM	Pearson	.a	1	0.828^**^	−0.205	0.076	0.956^**^	−0.077	0.282	0.025
	p (bilat)			0.000	0.447	0.779	0.000	0.778	0.290	0.925
	N	6	16	16	16	16	16	16	16	16
DS	Pearson	.a	0.828^**^	1	−0.002	0.025	0.956^**^	0.013	0.554^*^	0.039
	p (bilat)		0.000		0.995	0.926	0.000	0.963	0.026	0.886
	N	6	16	16	16	16	16	16	16	16
SM	Pearson	.a	−0.205	−0.002	1	0.594^*^	−0.108	0.900^**^	0.048	−0.296
	p (bilat)		0.447	0.995		0.015	0.691	0.000	0.860	0.266
	N	6	16	16	16	16	16	16	16	16
SS	Pearson	.a	0.076	0.025	0.594^*^	1	0.053	0.886^**^	−0.103	−0.351
	p (bilat)		0.779	0.926	0.015		0.845	0.000	0.705	0.183
	N	6	16	16	16	16	16	16	16	16
DA	Pearson	.a	0.956^**^	0.956^**^	−0.108	0.053	1	−0.033	0.438	0.034
	p (bilat)		0.000	0.000	0.691	0.845		0.902	0.090	0.901
	N	6	16	16	16	16	16	16	16	16
SA	Pearson	.a	−0.077	0.013	0.900^**^	0.886^**^	−0.033	1	−0.028	−0.361
	p (bilat)		0.778	0.963	0.000	0.000	0.902		0.918	0.169
	N	6	16	16	16	16	16	16	16	16
Self-esteem	Pearson	.a	0.282	0.554^*^	0.048	−0.103	0.438	−0.028	1	−0.209
	p (bilat)		0.290	0.026	0.860	0.705	0.090	0.918		0.437
	N	6	16	16	16	16	16	16	16	16
Creativity	Pearson	.a	0.025	0.039	−0.296	−0.351	0.034	−0.361	−0.209	1
	p (bilat)		0.925	0.886	0.266	0.183	0.901	0.169	0.437	
	N	6	16	16	16	16	16	16	16	16

^**^Significant correlation at 0.01 level (bilateral); ^*^Significant correlation at 0.05 level (bilateral).

a. It couldn't be calculated because at least one of the variables was constant.

DM: Deep motivation; DS: Deep strategy; SM: superficial motivation; SS: superficial strategy; DA: Deep learning approach; SA: superficial learning approach.

**Table 7:** Pearson's correlations between achievement, learning approaches, self-esteem and creativity for experimental group in posttest measures.

		**Achievement**	**DM**	**DS**	**SM**	**SS**	**DA**	**SA**	**Self-esteem**	**Creativity**
Achievement	Pearson	1	−0.780	−0.690	0.283	−0.076	−0.808	0.069	−0.610	−0.730
	p (bilat)		0.067	0.129	0.587	0.886	0.052	0.896	0.198	0.270
	N	6	6	6	6	6	6	6	6	4
DM	Pearson	−0.780	1	0.713^**^	−0.492	−0.247	0.936^**^	−0.397	0.489	−0.083
	p (bilat)	0.067		0.002	0.053	0.357	0.000	0.128	0.055	0.779
	N	6	16	16	16	16	16	16	16	14
DS	Pearson	−0.690	0.713^**^	1	−0.435	−0.244	0.914^**^	−0.367	0.658^**^	−0.004
	p (bilat)	0.129	0.002		0.092	0.362	0.000	0.163	0.006	0.989
	N	6	16	16	16	16	16	16	16	14
SM	Pearson	0.283	−0.492	−0.435	1	0.659^**^	−0.503^*^	0.899^**^	−0.492	−0.158
	p (bilat)	0.587	0.053	0.092		0.006	0.047	0.000	0.053	0.588
	N	6	16	16	16	16	16	16	16	14
SS	Pearson	−0.076	−0.247	−0.244	0.659^**^	1	−0.265	0.922^**^	−0.225	−0.035
	p (bilat)	0.886	0.357	0.362	0.006		0.321	0.000	0.402	0.906
	N	6	16	16	16	16	16	16	16	14
DA	Pearson	−0.808	0.936^**^	0.914^**^	−0.503^*^	−0.265	1	−0.414	0.613^*^	−0.052
	p (bilat)	0.052	0.000	0.000	0.047	0.321		0.111	0.011	0.860
	N	6	16	16	16	16	16	16	16	14
SA	Pearson	0.069	−0.397	0.367	0.899^**^	0.922^**^	−0.414	1	−0.385	−0.102
	p (bilat)	0.896	0.128	0.163	0.000	0.000	0.111		0.141	0.730
	N	6	16	16	16	16	16	16	16	14
Self-esteem	Pearson	−0.610	0.489	0.658^**^	−0.492	−0.225	0.613^*^	−0.385	1	−0.030
	p (bilat)	0.198	0.055	0.006	0.053	0.402	0.011	0.141		0.918
	N	6	16	16	16	16	16	16	16	14
Creativity	Pearson	−0.730	−0.083	−0.004	−0.158	−0.035	−0.052	−0.102	−0.030	1
	p (bilat)	0.270	0.779	0.989	0.588	0.906	0.860	0.730	0.918	
	N	4	14	14	14	14	14	14	14	14

^**^Significant correlation at 0.01 level (bilateral); ^*^Significant correlation at 0.05 level (bilateral).

DM: Deep motivation; DS: Deep strategy; SM: superficial motivation; SS: superficial strategy; DA: Deep learning approach; SA: superficial learning approach.

The results of the control group revealed negative and significant correlations between achievement and creativity in the pretest measures, without other significant relationships between the other variables (see Table 8). Nevertheless, in the posttest measures' results (see Table 9) creativity didn't correlate significantly with other variables, while self-esteem did. Specifically, self-esteem correlated positively with DS and DA, and negatively with superficial motivation (SM).

**Table 8:** Pearson's correlations between achievement, learning approaches, self-esteem and creativity for control group in pretest measures.

		**Achievement**	**DM**	**DS**	**SM**	**SS**	**DA**	**SA**	**Self-esteem**	**Creativity**
Achievement	Pearson	1	0.612	0.005	−0.378	0.207	0.412	−0.046	0.175	−0.990^**^
	p (bilat)		0.197	0.993	0.461	0.693	0.417	0.931	0.740	0.000
	N	6	6	6	6	6	6	6	6	6
DM	Pearson	0.612	1	−0.062	−0.806^*^	−0.496	0.625	−0.790^*^	0.360	−0.706
	p (bilat)	0.197		0.884	0.016	0.211	0.098	0.020	0.381	0.050
	N	6	8	8	8	8	8	8	8	8
DS	Pearson	0.005	−0.062	1	0.136	0.012	0.741^*^	0.076	−0.205	−0.037
	p (bilat)	0.993	0.884		0.748	0.977	0.036	0.859	0.626	0.932
	N	6	8	8	8	8	8	8	8	8
SM	Pearson	−0.378	−0.806^*^	0.136	1	0.143	−0.436	0.598	−0.622	0.325
	p (bilat)	0.461	0.016	0.748		0.735	0.280	0.118	0.099	0.433
	N	6	8	8	8	8	8	8	8	8
SS	Pearson	0.207	−0.496	0.012	0.143	1	−0.324	0.879^**^	−0.070	0.227
	p (bilat)	0.693	0.211	0.977	0.735		0.433	0.004	0.869	0.589
	N	6	8	8	8	8	8	8	8	8
DA	Pearson	0.412	0.625	0.741^*^	−0.436	−0.324	1	−0.473	0.082	−0.504
	p (bilat)	0.417	0.098	0.036	0.280	0.433		0.237	0.847	0.203
	N	6	8	8	8	8	8	8	8	8
SA	Pearson	−0.046	−0.790^*^	0.076	0.598	0.879^**^	−0.473	1	−0.357	0.340
	p (bilat)	0.931	0.020	0.859	0.118	0.004	0.237		0.386	0.410
	N	6	8	8	8	8	8	8	8	8
Self-esteem	Pearson	0.175	0.360	−0.205	−0.622	−0.070	0.082	−0.357	1	0.057
	p (bilat)	0.740	0.381	0.626	0.099	0.869	0.847	0.386		0.893
	N	6	8	8	8	8	8	8	8	8
Creativity	Pearson	−0.990^**^	−0.706	−0.037	0.325	0.227	−0.504	0.340	0.057	1
	p (bilat)	0.000	0.050	0.932	0.433	0.589	0.203	0.410	0.893	
	N	6	8	8	8	8	8	8	8	8

^**^Significant correlation at 0.01 level (bilateral); ^*^Significant correlation at 0.05 level (bilateral).

DM: Deep motivation; DS: Deep strategy; SM: superficial motivation; SS: superficial strategy; DA: Deep learning approach; SA: superficial learning approach.

**Table 9:** Pearson's correlations between achievement, learning approaches, self-esteem and creativity for control group in posttest measures.

		**Achievement**	**DM**	**DS**	**SM**	**SS**	**DA**	**SA**	**Self-esteem**	**Creativity**
Achievement	Pearson	1	0.642	0.003	−0.553	−0.178	0.403	−0.414	0.508	−0.518
	p (bilat)		0.169	0.996	0.256	0.736	0.428	0.415	0.303	0.293
	N	6	6	6	6	6	6	6	6	6
DM	Pearson	0.642	1	0.689^*^	−0.658	−0.439	0.932^**^	−0.611	0.662	−0.238
	p (bilat)	0.169		0.040	0.054	0.237	0.000	0.080	0.052	0.537
	N	6	9	9	9	9	9	9	9	9
DS	Pearson	0.003	0.689^*^	1	−0.672^*^	−0.768^*^	0.905^**^	−0.797^*^	0.702^*^	−0.017
	p (bilat)	0.996	0.040		0.047	0.016	0.001	0.010	0.035	0.964
	N	6	9	9	9	9	9	9	9	9
SM	Pearson	−0.553	−0.658	−0.672^*^	1	0.627	−0.723^*^	0.907^**^	−0.694^*^	0.065
	p (bilat)	0.256	0.054	0.047		0.071	0.028	0.001	0.038	0.868
	N	6	9	9	9	9	9	9	9	9
SS	Pearson	−0.178	−0.439	−0.768^*^	0.627	1	−0.642	0.897^**^	−0.381	−0.267
	p (bilat)	0.736	0.237	0.016	0.071		0.062	0.001	0.312	0.488
	N	6	9	9	9	9	9	9	9	9
DA	Pearson	0.403	0.932^**^	0.905^**^	−0.723^*^	−0.642	1	−0.758^*^	0.740^*^	−0.149
	p (bilat)	0.428	0.000	0.001	0.028	0.062		0.018	0.023	0.703
	N	6	9	9	9	9	9	9	9	9
SA	Pearson	−0.414	−0.611	−0.797^*^	0.907^**^	0.897^**^	−0.758^*^	1	−0.600	−0.107
	p (bilat)	0.415	0.080	0.010	0.001	0.001	0.018		0.087	0.784
	N	6	9	9	9	9	9	9	9	9
Self-esteem	Pearson	0.508	0.662	0.702^*^	−0.694^*^	−0.381	0.740^*^	−0.600	1	−0.311
	p (bilat)	0.303	0.052	0.035	0.038	0.312	0.023	0.087		0.416
	N	6	9	9	9	9	9	9	9	9
Creativity	Pearson	−0.518	−0.238	−0.017	0.065	−0.267	−0.149	−0.107	−0.311	1
	p (bilat)	0.293	0.537	0.964	0.868	0.488	0.703	0.784	0.416	
	N	6	9	9	9	9	9	9	9	9

^**^Significant correlation at 0.01 level (bilateral); ^*^Significant correlation at 0.05 level (bilateral).

DM: Deep motivation; DS: Deep strategy; SM: superficial motivation; SS: superficial strategy; DA: Deep learning approach; SA: superficial learning approach.

## 4 Discussion and conclusions

The aim of this study was to evaluate the Sakonduz curricular enrichment program by considering psychological aspects and academic achievement.

On the one hand, we expected an improvement in the academic achievement of the students. The experimental group maintained the median score in academic achievement while the control group decreased. But in both cases the differences were not significant. Therefore, the Sakonduz program did not improve nor worsen the academic achievement of the participants in the experimental group, so that the first hypothesis wasn't fulfilled. Nevertheless, in previous research it has been found that differentiated educational response based on curricular enrichment programs, taking into account the high abilities of schoolchildren, may improve the adaptation and the learning process in the school context, and the academic achievements of these students by comparing with those highly able students who didn't receive a response to their educational needs (García-Perales and Almeida, 2019). Also, this study refers to pre-study or pilot study, a first approximation of the curricular program's implementation in which a much smaller scale than the full curriculum (e.g., fewer students, less costly technology, fewer classes) has been considered but still preserving the essence of the program. A new curriculum program, once modifications have been included, needs to be launched as a full curriculum program (Nchindila and Corrigan, 2019). Thus, it is expected that full and long-term interventions in larger samples would lead toward better and more representative results.

On the other hand, in respect of the learning approach, we hypothesize that the Sakonduz program will contribute to the increase of a deep learning approach and the decrease of a superficial learning approach. The control group didn't show any changes, as expected, while the experimental group maintained the Deep Motivation Approach (DM) and decreased the Deep Strategy approach (DS), and consequently the Deep Learning General Approach (DA), with large effect sizes, contradicting the second hypothesis. We didn't obtain reliable results for the dimensions included in the superficial approach (SM, SS and SA). Therefore, we can infer that the program had a partial effect on the learning approach. We didn't find evidence of similar studies considering learning approach as a variable to measure a curricular program's effectiveness, but to understand this result, we could mention students with a low level of cognitive sophistication often tend to resort to a superficial approach (Hernández-Pina et al., 2004), and that this leads us to expect that the higher the cognitive complexity, the more tendence should be to use a deep and motivation approach. Nevertheless, a long-term intervention in larger samples should be carried out to go further from this pilot study and to obtain more conclusive results.

As for one of the psychological aspects, self-esteem, we hypothesize that the Sakonduz program will improve their self-esteem. The results show a considerable decrease in the level of self-esteem of the control group, with a large effect size, while it was maintained at the same high level in the experimental group. Therefore, the third hypothesis is not fulfilled, but still the Sakonduz program may have a preventive effect on the self-esteem of the experimental group. Considering the short-term of the program and the contextual conditions in which this was carried out, the immediate preventive impact of self-esteem can be considered as a promising result. This is in coherence with previous findings that enhance the positive effect of educational responses in the self-esteem of highly able students (Hertzog, 2003). In any case, we should mention that we didn't find evidence of similar studies considering self-esteem as a variable to measure a curricular program's effectiveness. Therefore, we believe that this could be considered as a contribution to the curricular educational programs' research field and that it should be expected that a long-term and full enrichment program would lead to the improvement of self-esteem, or at least to maintain a high level of self-esteem as we have found in this study.

The fourth hypothesis predicted that the Sakonduz program will foster the development of creativity of students. But creativity measurements didn't have consistent results. In the control group creativity decreased, but not significantly, while for the experimental group the measure didn't obtain good reliability results. Therefore, we cannot infer the impact of the program on participants' creativity level. In any case, previous literature enhances the role of enrichment programs to foster creativity (Reis et al., 2021). Therefore, a long-term program should be expected to have a greater impact on creativity productivity, as previous research has stated.

Finally, the relationships between academic achievement, learning approach, self-esteem and creativity were analyzed. We hypothesize that the Sakonduz program will show a positive relationship between academic achievement, deep learning approach, self-esteem and creativity and a negative relationship between the previous variables and superficial learning approach. This hypothesis has been partially confirmed. In the experimental group a positive and significant relationship was found between deep strategies and self-esteem in the pre- and posttest measures, and also with deep learning approach in the posttest. But deep motivation, academic achievement and creativity didn't show any significant relationship. Negative correlations were not significant in any case. In the control group, similar results were found, with positive correlations between DS and DA, and self-esteem in the posttest measure, and negative correlation between SM and self-esteem. These unexpected results bring us to the question of what kind of learning would be effective to foster changes in academic achievement, motivation, and creativity. In this sense, (Rabello-Mestre et al. 2025) propose creative learning as a practice that could consider students' affective demands and ways of knowing that can complement critical engagement. They define creative learning as a learner-driven process that emphasizes epistemic agency, interaction, and direct creative engagement with the subject matter. It is an eclectic approach that could foster motivation and creativity, and consequently, academic performance by mobilizing affective, sensory, and intellectual resources to produce new and meaningful understandings for the learner. It would be interesting to analyze in further studies whether the learning processes developed in the Sakonduz program (or others) include this new paradigm of the relationship between learning and creativity.

This study has not been carried out without limitations. The most important barrier to obtaining more conclusive results has been the high experimental mortality. In the experimental group, 65.96% of the participants abandoned the study; in the control group, 64.00% of participants. One of the factors that may have influenced the abandonment is the lack of appropriate involvement of the teachers and the school in the study. In fact, in the experimental group there were several schools that had irregular monitoring of the program. The experimental mortality or participants drop out is a common phenomenon in longitudinal or repeated measures studies. Several factors may influence, but in this case, the differential attrition seems to be the most significant. This occurs when participants drop out of the study for reasons that are related to the treatment, and when the degree of attrition differs between the intervention and control conditions, being related to perceived efficacy or tolerability of the intervention (Crutzen et al., 2015). To our knowledge, there is no evidence of differential attrition in studies evaluating educational programs; therefore, greater research should be developed to obtain such evidence and to know if our study's results may be comparable to others. Furthermore, the small sample size could interfere with the results of this study. Considering these important limitations, it should be seen whether with a full, more intense and long-term interventions in larger samples the trends would be maintained or not. Also, educational agents' implications are crucial for the successful development of an enrichment program and for its impact on psychological aspects. In this line, Reis et al. (2021) manifest that those teachers who implement adequate enrichment programs and pedagogy foster opportunities to learn, to advance in contents, processes and products, and promote the access to new ideas and broader interdisciplinary contents. Moreover, these teachers also foster effective independent and autonomous learning and contribute to the development of creativity. Another limitation of the study is the unequal size of subsamples, because the small group is smaller than the experimental group. This could be understood if we consider that the control group wasn't participating in the program and they would lack of motivation to fulfill with all requirements of the study. The experimental mortality in this group was also high. Therefore, for future studies, other strategies to engage participants should be considered.

In conclusion, the Sakonduz curricular enrichment program has been developed to offer highly able students an opportunity to learn and to engage in their educational processes at school. This program should be improved and implemented as a long-term and more intense full curricular program to obtain more evidence of its effect in psychological aspects such as learning approach, self-esteem and creativity. Nevertheless, it would be important the implication of the schools and teachers to implement the program correctly and to facilitate the highly able students a program through which they could obtain an appropriate educational response to their needs.

## Data Availability

The raw data supporting the conclusions of this article will be made available by the authors, without undue reservation.
